# A Case of Stanford Type A Aortic Dissection Presenting as Syncope and Neurologic Deficits Without Pain: Diagnostic Pitfalls and a Therapeutic Dilemma

**DOI:** 10.7759/cureus.87979

**Published:** 2025-07-15

**Authors:** Umar Ismail

**Affiliations:** 1 Medicine, NHS Wales, Wales, GBR

**Keywords:** aad, lactate, painless aortic dissection, shock, ’stanford type a acute aortic dissection, ste-acs, stroke, type a aortic dissection

## Abstract

Acute aortic dissection (AAD) is a true medical emergency that classically presents with sudden severe tearing chest pain that may radiate to the back or with tearing abdominal pain. When it presents atypically without pain, diagnostic delays or misdiagnosis are common, often with devastating consequences. We report the case of a 59-year-old male with uncontrolled hypertension who first presented to an outside emergency department (ED) with multiple collapses and was discharged after assessment. He presented 48 hours later to our ED following collapse and loss of consciousness. The patient was in shock upon arrival. Transient right upper limb weakness and slurring of speech were noted on initial assessment. Acute coronary syndrome (ACS) with cardiogenic shock was suspected on the basis of electrocardiographic (ECG) changes and raised troponin, and ACS treatment was administered. However, bedside echocardiogram performed to assess left ventricular function as part of the ACS work-up suggested Stanford type A AAD, which was confirmed by computed tomogram (CT) of the aorta. Emergency surgical repair of the aorta was performed after transfer to a tertiary hospital, with good postoperative recovery. This case highlights the importance of maintaining a high index of suspicion for aortic dissection in patients presenting with syncope and neurological deficits even in the absence of classical symptoms. Simulation training specifically tailored to scenarios of atypical presentations of AAD may be of benefit to emergency clinicians and help reduce the unacceptably high rate of misdiagnosis.

## Introduction

Acute aortic dissection (AAD) is one of the most catastrophic clinical syndromes that involves the aorta and is a true medical emergency. It classically presents with acute severe chest, back, or tearing abdominal pain. However, 5-17% of patients present atypically with minimal or no pain, making diagnosis challenging [1-3]. Painless presentations tend to have complex clinical manifestations involving multiple organs and consequently have a high rate of delayed or missed diagnosis and poor outcomes. In a 2022 systematic review including 1,663 patients, Lovatt et al. reported an overall rate of misdiagnosis of 33.8% [1]. Factors related to misdiagnosis included the presence of symptoms and features associated with other diseases such as acute coronary syndrome (ACS), stroke, or pulmonary embolism. Misdiagnosis not only causes a delay in establishing an accurate diagnosis and starting treatment but may also result in the administration of therapies that could have disastrous consequences. Patients with strokes secondary to AAD who received intravenous thrombolysis have been reported in the literature [3].

AAD has a reported mortality rate of 40% at initial presentation, which increases by 0.5-1% every hour during the first 48 hours after the onset of symptoms if not addressed promptly [4,5]. In a 2004 study conducted on the International Registry of Aortic Dissection (IRAD) data, Park et al. reported a prevalence of 6.4% for painless AAD [6]. In the study, common presentations of painless AAD included syncope (33.9%), new-onset neurological deficit (23.7%), stroke (11.3%), congestive heart failure (19.7%), coma or spinal cord ischemia (17.0%), acute renal failure (13.6%), myocardial infarction (7.1%), and mesenteric ischemia (6.8%). Up to 70% of patients with painless AAD have either a transient or persistent disturbance of consciousness [4].

The most commonly used classification for AAD in clinical practice is the Stanford classification [5]. This defines type A AAD as involving the ascending aorta, potentially extending to the aortic arch and thoracoabdominal aorta. Type B affects the descending thoracic or thoracoabdominal aorta beyond the origin of the left subclavian artery, sparing the ascending aorta. Painless presentations are more common in type A than in type B dissections and are linked to higher mortality [6]. Risk factors for AAD include smoking, hypertension, bicuspid aortic valve, and underlying connective tissue diseases such as Marfan syndrome or Ehlers-Danlos syndrome [5]. Both smoking and connective tissue disease lead to structural weakening of the aortic tunica media.

The core pathophysiologic principle underlying AAD is an increase in pressure against a structurally weak aortic wall and shearing force from blood flow, leading to increased wall tension [5]. These eventually result in tearing of the intima, which allows blood to enter and separate the layers of the tunica media, creating a false lumen within the aortic wall. The creation of a false lumen has at least two consequences. First, the false lumen can propagate and lead to stretching of nociceptors in the media and adventitia, resulting in pain often described as a "tearing" or "ripping.". Second, other vessels that originate from the aorta can have their origins compromised by arising from within the false lumen, leading to ischemia of the end organs supplied by these vessels. This is responsible for the wide-ranging manifestations seen in AAD as multiple vessels supplying different organs can be affected all at once. Patients experience varying degrees of pain depending on the acuity of medial tearing and its speed of progression. Rapid expansion is more likely to cause stimulation of mechanoreceptors and pain compared to a more gradual process, and some patients may not experience pain at all.

## Case presentation

A 59-year-old previously healthy male presented to our ED via ambulance following collapse at home during which he lost consciousness for 10 minutes. Upon regaining consciousness, he exhibited slurred speech and weakness in the right upper limb, which resolved by the time of arrival to the ED. The patient was a heavy smoker (20 cigarettes/day) with a history of unmedicated hypertension. There was no family history of connective tissue or cardiovascular disease.

Interestingly, the patient was seen in another ED 48 hours prior to this presentation following multiple episodes of collapse. Transient ischemic attack (TIA) was diagnosed at that presentation after evaluation with CT of the brain, and he was discharged with no concerning findings on complete recovery. On arrival at our ED, he was in hypotensive shock with a systolic blood pressure of 79 mmHg, pulse rate of 105 beats per minute, and oxygen saturation of 100% on room air. Clinical signs of left leg ischemia (pallor and mottling) were noted, and admitting lactate was 4 mmol/L, further suggesting tissue ischemia, although this was not appreciated initially. Other systemic examinations were stable, and there was no focal neurology. ECG showed what was believed to be anterior ST-segment elevation without reciprocal changes. Although blood cell counts were generally normal, platelets were significantly low, suggesting possible consumption. The metabolic panel showed moderate acute kidney injury, and elevated transaminases and troponin T (Table 1), all in keeping with widespread end-organ ischemia. Although all of these findings have been noted and suggested the likely diagnosis, the connection was not made. The rest of admitting investigations are summarized in Table 1.

**Table 1:** Initial lab findings on admission AST, aspartate transaminase; ALT, alanine transaminase; ALP, alkaline phosphatase; MCV, mean corpuscular volume

Lab	Value	Reference range
Metabolic profile		
Creatinine	163 mmol/L	58-110 mmol/L
Urea	8.7 mmol/L	2.5-7.8 mmol/L
AST	350 U/L	<50 U/L
ALT	514 U/L	<50 U/L
ALP	162 U/L	30-130 U/L
Bilirubin	29 umol/L	<21 umol/L
Albumin	35 g/L	35-55 g/L
Calcium	2.16 mmol/L	2.20-2.60 mmo/L
Troponin T	190 ng/L	<14 ng/L
Lactate	4.0mmol/L	0.5-1.6 mmol/L
Full blood count		
Hemoglobin	125 g/L	130-180 g/L
MCV	88 fL	80-100 fL
Platelets	69 K/uL	150-400 K/uL
White cells	9.9 K/uL	4.0-11.0 K/uL
Neutrophils	8.1 K/uL	1.7-7.5 K/uL

**Figure 1:**
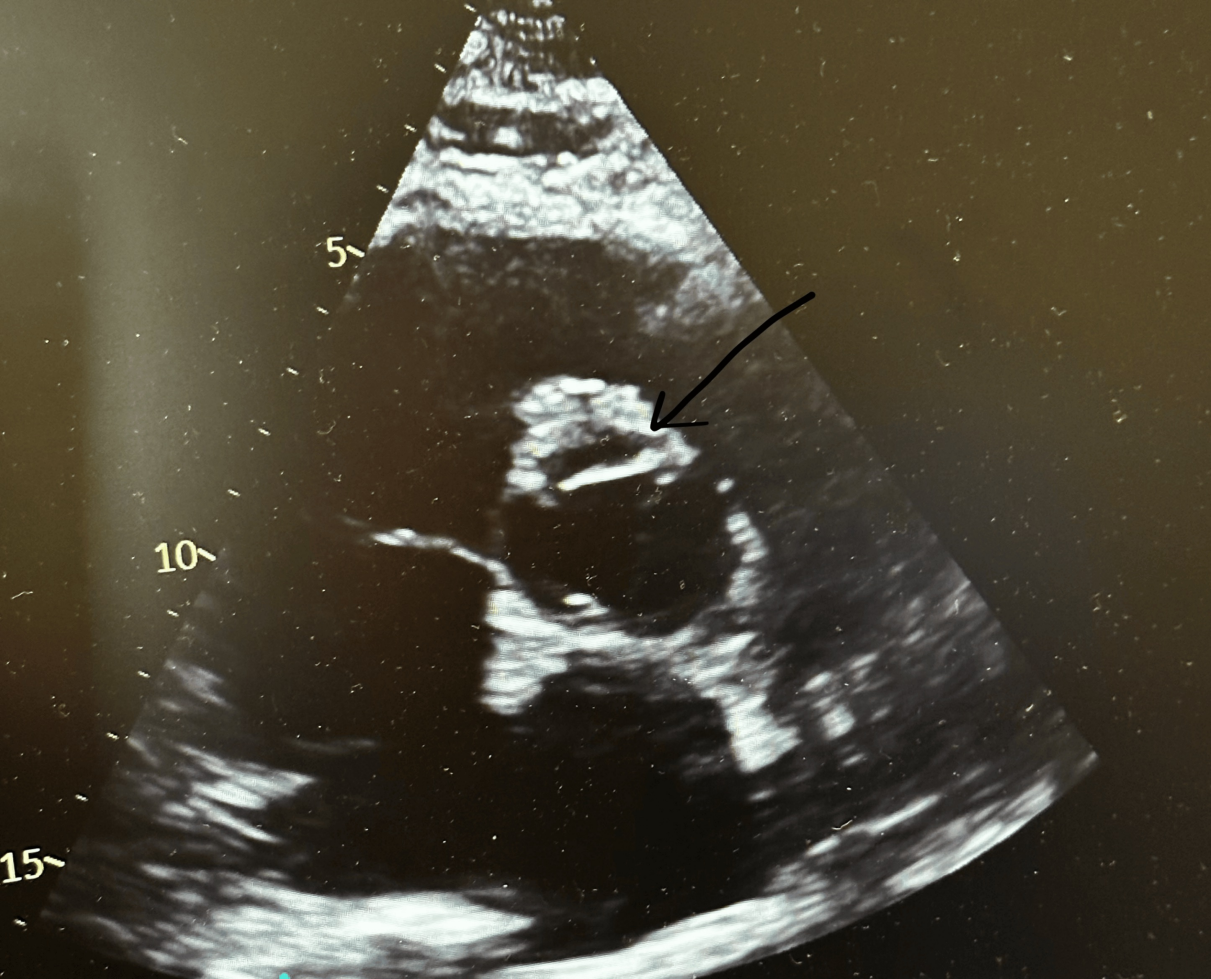
Transthoracic echocardiogram showing the dissecting flap in the aortic root (black arrow) in keeping with Stanford A dissection.

Given the initial hypotension, elevated lactate level, ECG changes, and troponin leak, a diagnosis of ST-elevation myocardial infarction (STEMI) with cardiogenic shock was made, and the patient was initially managed with dual anti-platelet therapy and low-molecular-weight heparin. An urgent transthoracic echocardiogram (ECHO) was requested to assess left ventricular function as part of ACS work-up, which revealed a dissecting flap in the proximal aorta (Figure 1), pericardial effusion, and reduced left ventricular ejection fraction. These ECHO findings were very concerning for a proximal aortic dissection extending into the aortic root and pericardium with potential hemopericardium.

**Figure 2:**
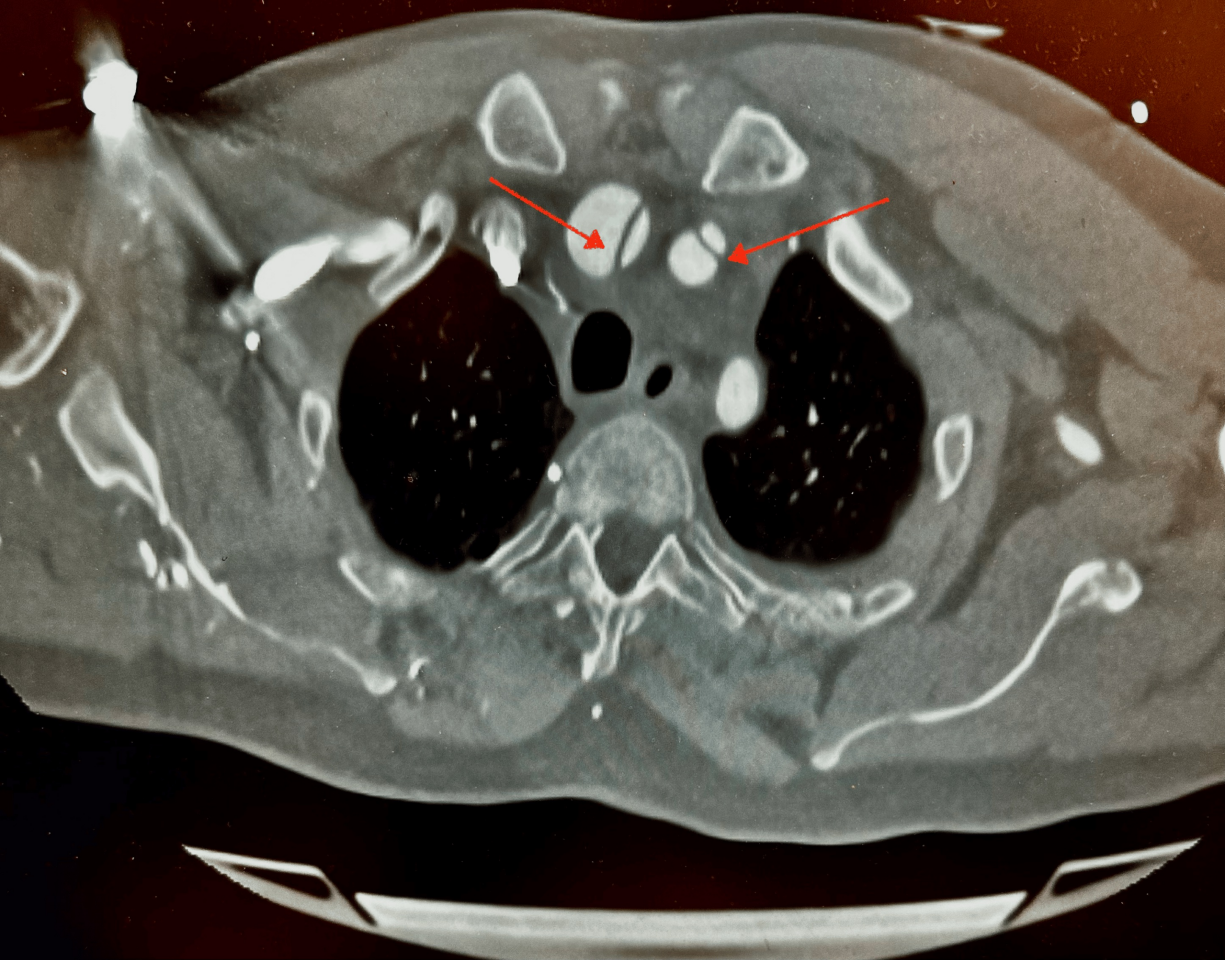
Dissecting flaps extending into the brachiocephalic and left common carotid arteries (red arrows), which provide a likely explanation for the patient’s neurological symptoms at presentation.

**Figure 3:**
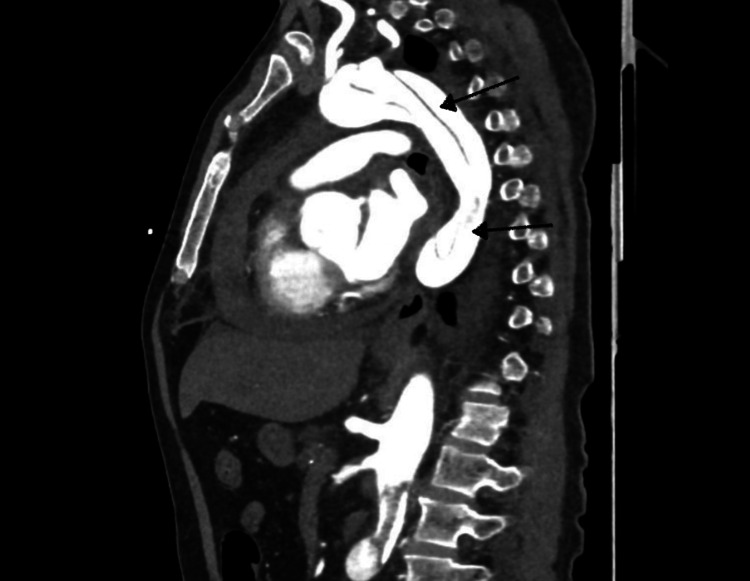
Sagittal view of CT aortogram showing Stanford type A AAD with the dissecting flap extending distally into the descending aorta.

**Figure 4:**
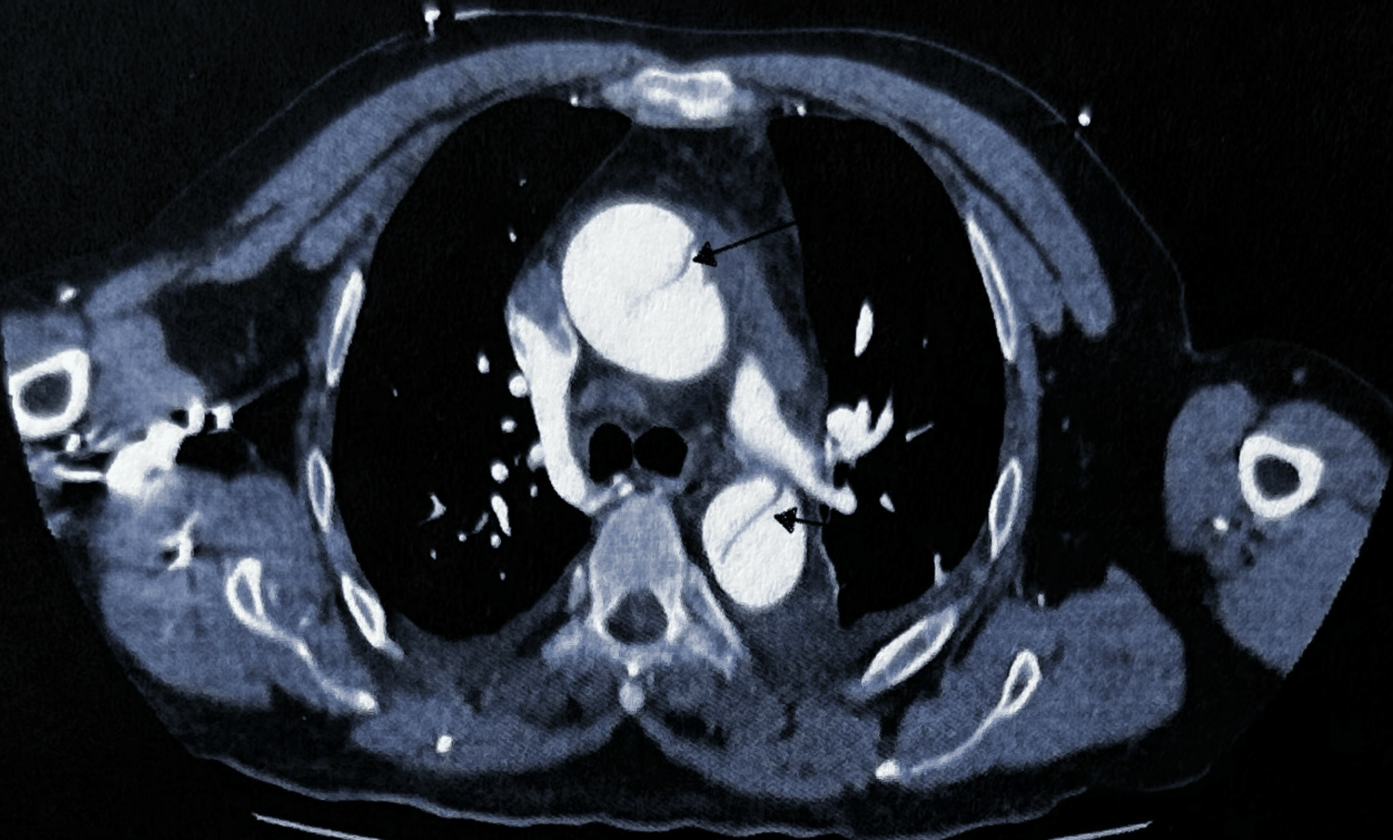
CT aortogram showing dissecting flaps in both the ascending and descending aorta.

**Figure 5:**
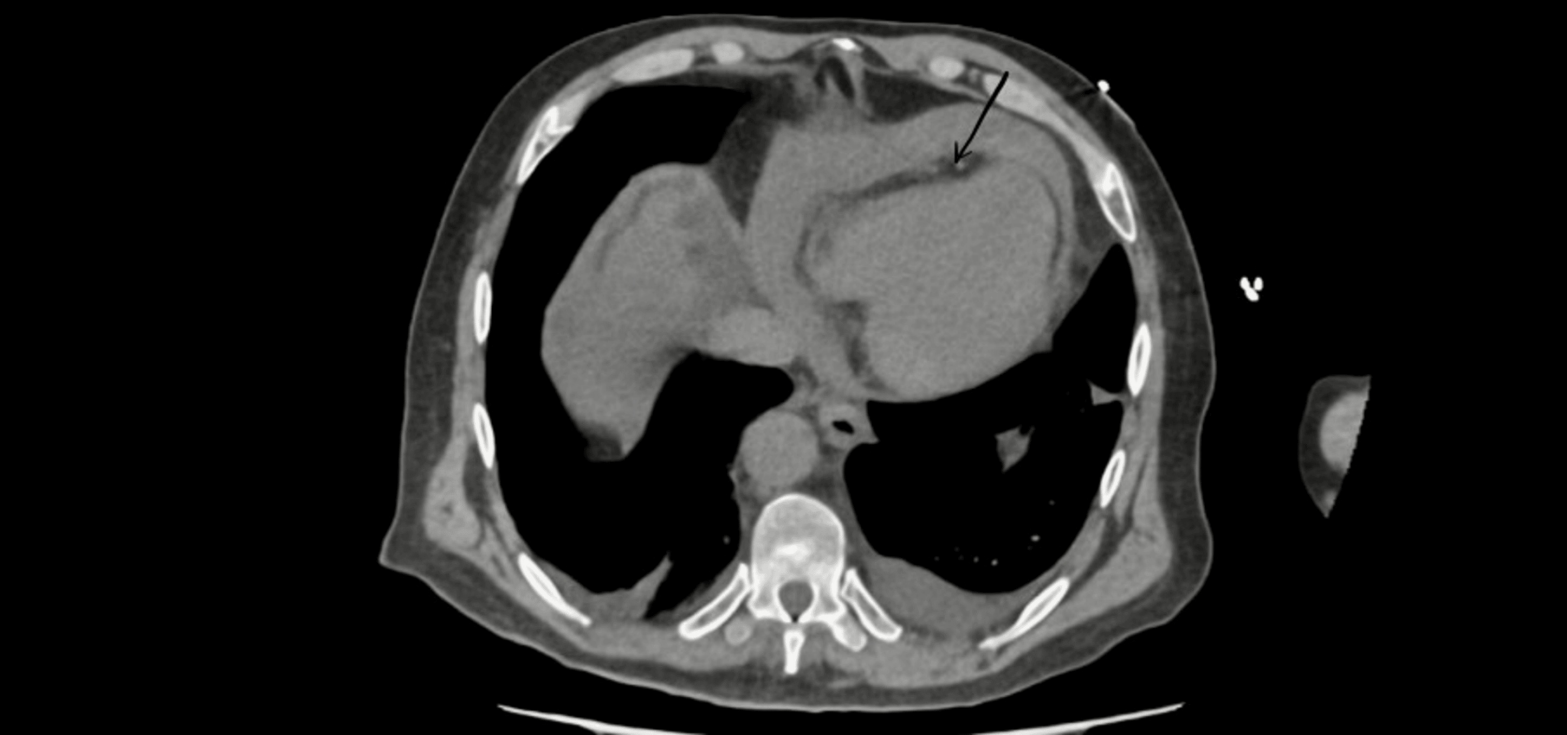
Pericardial effusion (black arrow) on CT scan. This was confirmed intraoperatively to be a hemopericardium.

CT aortogram was immediately requested, which confirmed Stanford type A AAD from the aortic root and arch, extending into the brachiocephalic and left common carotid arteries (Figure 2), and distally into the abdominal aorta up to the bifurcation of the common iliac arteries and beyond with partial occlusion of the left femoral artery (Figure 3) and (Figure 4). Moderate pericardial effusion was also noted on the CT (Figure 5). The patient was transferred to our tertiary center, where he underwent emergency ascending aortic repair and hemi-arch replacement. Postoperative recovery was complicated by myocardial infarction and prolonged hospital stay, but the patient survived and was discharged with good functional outcome (independent); he was doing well on follow-up. Left ventricular function subsequently normalized.

## Discussion

This case highlights the diagnostic challenges and treatment dilemma often encountered in suspected painless AAD in a typical ED setting [1-6]. Physicians are often under intense pressure to make a rapid diagnosis in a crowded ED to facilitate patient flow, often at the expense of careful consideration of symptoms, signs, and investigations that would ensure accurate diagnosis. There is a high tendency to resort to heuristics and mental shortcuts.

It is important that clinicians always consider all the relevant past and present information about a patient to look for important clues to develop the right differential diagnoses in challenging clinical presentations. Although this seems obvious, sometimes clinicians tend to neglect important information because of anchoring and heuristic biases, which lead to medical errors with critical implications [7]. One potentially fatal disease can mimic the clinical presentation of another, and with both conditions requiring time critical interventions and having radically different treatment approaches, outcomes could be catastrophic [8]. In some cases, multiple pathologies may run concurrently, and one process may entirely be driven by another. Identifying the unifying diagnosis is key to delivering the right treatment.

Cognitive biases are very common in clinical practice and appeared to have played a significant role in the management decisions examined in this presentation. In the first clinical encounter in the outside ED, the clinicians anchored onto an intracranial cause to explain the patient symptoms. This is evidenced by the decision to pursue a non-contrasted CT of the brain and discharge the patient once all symptoms resolved - this is premature closure, a type of clinical bias. The presentation with transient loss of consciousness was likely a missed opportunity to diagnose the AAD, which was misdiagnosed as a TIA. In patients with AAD who present with neurological symptoms, diagnosis is sometimes missed due to either a lack of physician awareness of the connection between the two or the sense of urgency to thrombolyze the stroke, thus preventing proper assessment [9].

In our ED, the clinician displayed multiple cognitive biases during the clinical encounter. First, the clinician anchored onto an ACS diagnosis probably due to the presumed "ECG changes," succumbing to availability bias likely because ACS is a diagnosis they were most familiar with. Once that happened, confirmation bias kicked in and they filtered out data that were not supportive of their diagnosis. This is not an indictment, it is the unfortunate reality of clinical practice in a high-stress, high-pressure environment. The misdiagnosis of ACS led to treatment with high-dose antiplatelets and low-molecular-weight heparin, which could have resulted in poor outcomes. While inappropriate anticoagulation may lead to immediate complications such as expansion of a hemopericardium and tamponade, some hemorrhagic complications may occur later, such as intraoperative bleeding that can severely impact the overall surgical risk. Our patient's initial ECHO showed evidence of pericardial collection, which was confirmed intra-operatively to be a large hemopericardium with features of peri-tamponade.

Interestingly, the connection between acute kidney injury, focal neurological deficit, raised lactate, raised transaminases, and left leg ischemic changes was not made initially and therefore did not coalesce into a unified diagnosis early. The patient only underwent ECHO to assess left ventricular function as part of work-up for ACS, which incidentally revealed the proximal aortic dissection and hemopericardium, which was then confirmed with CT aortogram. Although our patient’s outcome was very favorable, it was a near-miss event that highlights how easily things can go wrong in the absence of careful contemplation of clinical signs and what they mean both separately and when put together. This potential makes it important to switch from a heuristic to a more analytical thinking model when developing differential diagnoses [10].

## Conclusions

Syncope and neurological deficits particularly when associated with hypotension and metabolic derangements should prompt consideration of AAD. Clinical evidence of ischemia affecting multiple organs in the absence of a clear cause such as severe sepsis should also raise suspicion. Clinicians should maintain a high index of suspicion in such scenarios. While early imaging with point-of-care ECHO and rapid CT aortogram is key to expediting diagnosis and interventions, broadening the list of differential diagnoses in complex clinical presentations like this case is even more critical.

While many hospitals already provide simulation training for critical emergencies such as cardiac arrest and shock for emergency clinicians, these sessions can be leveraged to simulate presentations specifically tailored to scenarios of atypical presentations of AAD, such as syncope with shock and neurological deficits, and may be of benefit in raising awareness among emergency clinicians to help reduce the high rate of misdiagnosis by training clinicians to expand their list of differential diagnosis and avoid premature closure in clinical decision-making.
